# Supplementary material for: Intrinsically disordered protein biosensor tracks the physical-chemical effects of osmotic stress on cells
Source: Nat Commun. 2021 Sep 14;12:5438. doi: 10.1038/s41467-021-25736-8 (PMC8440526; doi:10.1038/s41467-021-25736-8)
Supplement: Supplementary file 1 — Supplementary Information [file 41467_2021_25736_MOESM1_ESM.pdf]

Supplementary Information

Intrinsically disordered protein biosensor tracks the physical-chemical effects of osmotic stress on cells

Cesar L Cuevas-Velazquez[1,2,3,4*], Tamara Vellosillo[1,2], Karina Guadalupe[5,6], H Broder Schmidt[7], Feng Yu[5,8], David Moses[5,6], Jennifer AN Brophy[1,2], Dante Cosio-Acosta[3], Alakananda Das[9], Lingxin Wang[9], Alexander M Jones[10], Alejandra A Covarrubias[3*], Shahar Sukenik[5,6,8*], José R Dinneny[1,2*]

[1] Department of Biology, Stanford University, Stanford, CA, 94305, USA
[2] Department of Plant Biology, Carnegie Institution for Science, Stanford, CA, 94305, USA
[3] Departamento de Biología Molecular de Plantas, Instituto de Biotecnología, Universidad Nacional Autónoma de México, Cuernavaca, Mor., 62210 Mexico
[4] Departamento de Bioquímica, Facultad de Química, Universidad Nacional Autónoma de México, Ciudad de México, 04510, Mexico
[5] Center for Cellular and Biomolecular Machines (CCBM), University of California, Merced, CA, 95343, USA
[6] Chemistry and Chemical Biology Program, University of California, Merced, CA, 95343, USA
[7] Department of Biochemistry, Stanford University School of Medicine, Stanford, CA, 94305, USA
[8] Quantitative Systems Biology Program, University of California, Merced, CA, 95343, USA
[9] Department of Molecular and Cellular Physiology, Stanford University, Stanford, CA, 94305, USA
[10] Sainsbury Laboratory, Cambridge University, Cambridge, CB2 1LR, UK
* Correspondence C.L.C.V. cuevas@quimica.unam.mx
* Correspondence A.C.R. alejandra.covarrubias@mail.ibt.unam.mx
* Correspondence S.S. ssukenik@ucmerced.edu
* Correspondence J.R.D. dinneny@stanford.edu

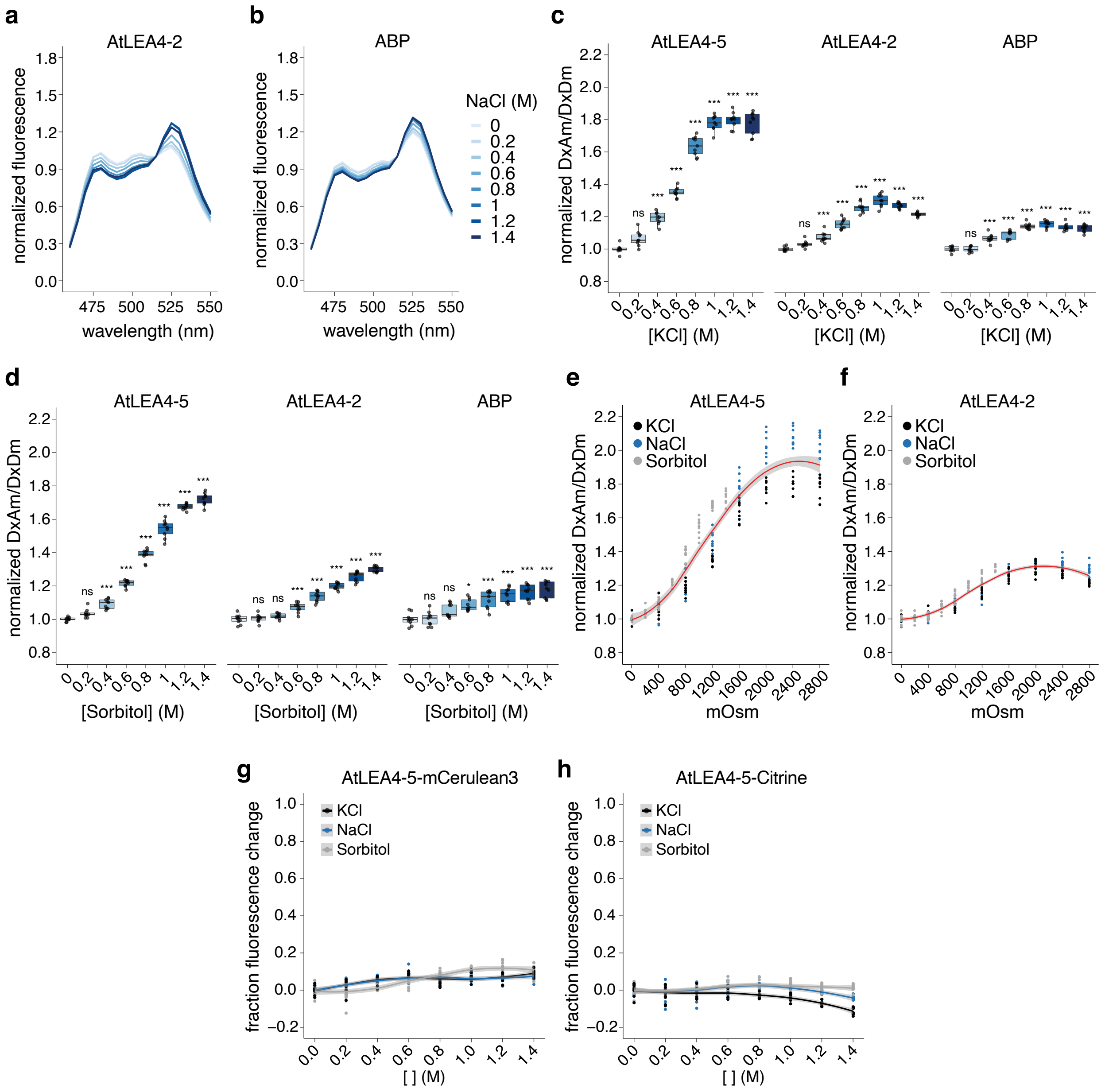

Supplementary Figure 1. (a) Fluorescence emission spectra of NaCl-treated live yeast cells expressing the biosensor construct using AtLEA4-2 as the sensory domain. Fluorescence values were normalized to the value at 515 nm. (b) Fluorescence emission spectra of NaCl-treated live yeast cells expressing the biosensor construct using ABP as the sensory domain. Fluorescence values were normalized to the value at 515 nm. (c) Normalized FRET ratio (DxAm/DxDm) of live yeast cells treated with different concentrations of KCl. Cells are expressing the biosensor construct using either AtLEA4-5, AtLEA4-2, or ABP as the sensory domain. n = 9 independent measurements. Two-way ANOVA. *$p < 0.05$, **$p < 0.01$, ***$p < 0.001$. Boxes represent 25th-75th percentile (line at median) with whiskers at 1.5*IQR. (d) Normalized FRET ratio (DxAm/DxDm) of live yeast cells treated with different concentrations of sorbitol. Cells are expressing the biosensor construct using either AtLEA4-5, AtLEA4-2, or ABP as the sensory domain. n = 9 independent measurements. Two-way ANOVA. *$p < 0.05$, **$p < 0.01$, ***$p < 0.001$. Boxes represent 25th-75th percentile (line at median) with whiskers at 1.5*IQR. (e) Normalized FRET ratio (DxAm/DxDm) of live yeast cells expressing the biosensor construct using AtLEA4-5 as the sensory domain. Cells were hyperosmotically shocked to the indicated osmolarity with either NaCl (blue), KCl (black), or sorbitol (gray). Continuous line was smoothed using R with a loess smoothing function. Shaded region indicates 95% confidence interval.

(f) Normalized FRET ratio (DxAm/DxDm) of live yeast cells expressing the biosensor construct using AtLEA4-2 as the sensory domain. Cells were hyperosmotically shocked to the indicated osmolarity with either NaCl (blue), KCl (black), or sorbitol (gray). Continuous line was smoothed using R with a loess smoothing function. Shaded region indicates 95% confidence interval. (g) Fraction fluorescence change relative to the standard condition of live yeast cells expressing AtLEA4-5-mCerulean3 (donor-only construct) when cells were exposed to different concentrations of NaCl (blue), KCl (black), or sorbitol (gray). Continuous lines were smoothed using R with a loess smoothing function. Shaded regions indicate 95% confidence interval. (h) Fraction fluorescence change relative to the standard condition of live yeast cells expressing AtLEA4-5-Citrine (acceptor-only construct) when cells were exposed to different concentrations of NaCl (blue), KCl (black), or sorbitol (gray). Continuous lines were smoothed using R with a loess smoothing function. Shaded regions indicate 95% confidence interval. Source Data are provided as a Source Data file.

a

Construct	Donor	Acceptor
FP1	t7.eCFP.t9	Aphrodite.t9
FP2	t7.TFP.t9	Aphrodite.t9
FP3	mTFP.t9	Aphrodite.t9
FP4	Cerulean	Aphrodite.t9
FP5	Cerulean	Citrine
FP6	edCerulean	edCitrine
FP7	t7.eCFP.t9	edAphrodite.t9
FP8	Citrine	mCerulean
FP9	edCerulean	edAphrodite.t9
FP10	mCerulean3	Citrine

b

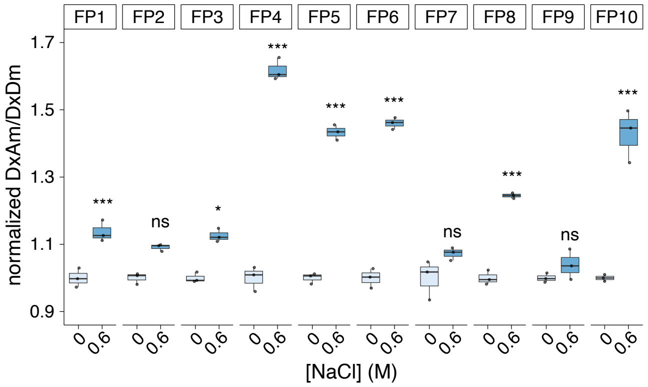

Supplementary Figure 2. (a) Different FRET pairs used in this study. FP: FRET pair. t7: 7 amino acid terminal truncation. t9: 9 amino acid terminal truncation. m: monomeric variant. ed: enhanced dimerization variant. (b) Normalized FRET ratio (DxAm/DxDm) change of live yeast cells expressing AtLEA4-5 fused to the different FRET pairs in (a), subjected to 0 M or 0.6 M NaCl. n = 3 independent experiments. Two-way ANOVA. *p < 0.05, **p < 0.01, ***p < 0.001. Boxes represent 25th-75th percentile (line at median) with whiskers at 1.5*IQR. Source Data are provided as a Source Data file.

a

AtLEA4-5

MQSMKETASN IAASAKSGMD KTKATLEEKA EKMKTRDPVQ KQMATQVKED
KINQAEMQKR ETRQHNAAMK EAAGAGTGLG LGTATHSTTG QVGHGTGTHQ
MSALPGHGTG QLTDRVVEGT AVTDPIGRNT GTGRTTAHNT HVGGGGATGY
GTGGGYTG

Scramble-1

VHDGETPVGP RTEQAMTTQE QEAKNTGGLA NIGAQTDTKK QTAQASHQTA
TEKTKAKGAT GRRTGGYGNN HGMVKMGVIM GSKGKEMGLR TDTMTTGHSS
GIGKQHLMAL RHAATMGTRA SSGVKGGETV THEPAAAGQG KGTNYLATTG
EQADVGTD

Scramble-2

GPSIKKTQTL KHPGNALEMT VGMAEGGNTV ARTHDSSGDS HQTAETKDHG
GTGAMYKLHK HNGKTQQVDT GMATAGAVPY AKEKTSTGSN ATAEHTTGKM
TGTAQIMETG RLAERVGMTI DARTGGLNGG GTARMQRKQT QGAEKAGVGQ
QAGVTGET

Scramble-3

GDTNGSTTAM RAQTDEKMAT RQKEAGNDRY TSGHTAGVTA KTLGLPDARS
KSGPKKSTET GEHQGTMEQY TTHGEGHGKA AQVIAATGLK TNTKQANGIK
KTLQRQMQTA VGAMNHEGEA AVALVGGHGE HAGTVVGGTQ GTGMTMSMGI
PTDKTGGR

Scramble-4

QGGMGKAHKG EQKGTVTTNI AVGAVTAGHQ TEGLEQGGHE ADTVAAMAKS
LQPKRTTTGT GNTRTLELSG HTAATNSMPK GTHEMGEMTA QGRIGTMVKL
GGYQSGTHGE DAQAKKAGDS GSTKRDNAGA RQIKAETKVG VMGTHTTTQD
RANYGPMT

Scramble-5

SHPAGMKATM GHQHEYQEQT SAMGKGTLEI EDTGTAAGEQ RTHDKASTGA
TTTGDQETMG GMQAKVKMHL LTAGLQAVGE AGATTMREHY GTHPTAGQNV
RPKGTTQGSK ADRILDTRMN SVKKAGGEIN GTNSGGGGTK VGAKTTNTGA
KGVRQATV

b

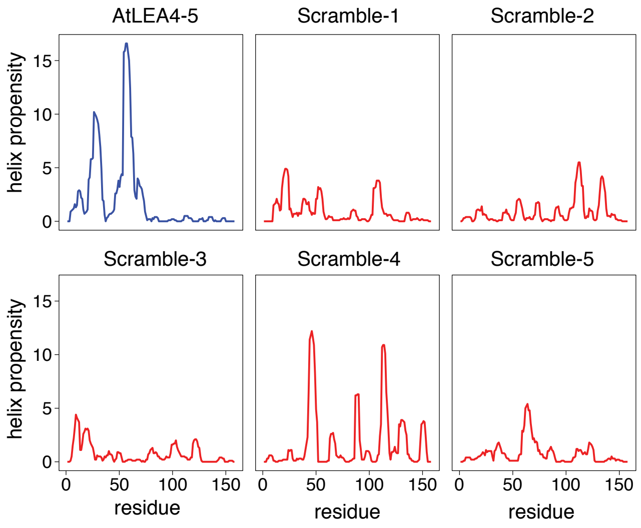

c

Protein	Length (aa)	Kappa	FCR	Omega (polar)	Omega (hydrophobic)
AtLEA4-5	158	0.061	0.203	0.214	0.186
Scramble-1	158	0.174	0.203	0.216	0.063
Scramble-2	158	0.164	0.203	0.225	0.099
Scramble-3	158	0.177	0.203	0.321	0.096
Scramble-4	158	0.186	0.203	0.272	0.104
Scramble-5	158	0.21	0.203	0.256	0.11

Supplementary Figure 3. (a) Amino acid sequence of AtLEA4-5 and the five different scrambled versions of AtLEA4-5 used in this study. (b) Agadir α-helix propensity (%) prediction of AtLEA4-5 (blue) and the five different scrambles of AtLEA4-5 (red). (c) CIDER parameters of AtLEA4-5 and the five different scrambled versions. Kappa: Measure of the extent of charged residues (R,K,E,D) segregation; FCR: Fraction of charged residues; Omega (polar): Measure of the extent of polar residues (Q,N,S,T,G,H,C) segregation; Omega (hydrophobic): Measure of the extent of polar residues (A,L,M,I,V) segregation. Source Data are provided as a Source Data file.

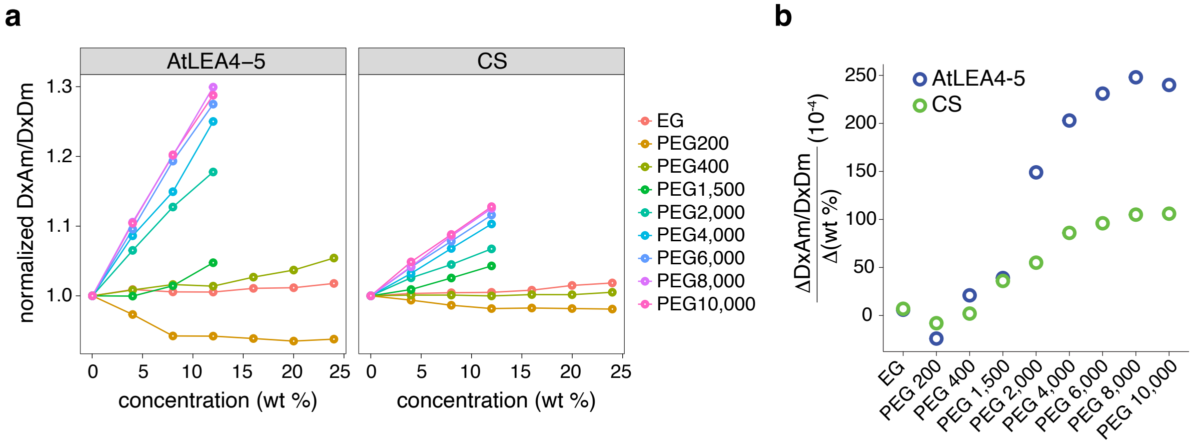

Supplementary Figure 4. (a) Normalized FRET ratio (DxAm/DxDm) of purified recombinant full-length AtLEA4-5 or CS biosensor constructs at different concentrations (wt %) of EG or the indicated molecular weight PEG. FRET ratio values of treatments with more than 12% high molecular weight PEGs are not shown because AtLEA4-5 and CS crashed out of solution, resulting in an uninterpretable FRET signal. EG: Ethylene glycol. PEG: Polyethylene glycol. Numbers represent the average molecular weight of each type of PEG. (b) FRET ratio rate of change (10^{-4}) as a function of different molecular weight osmolytes concentration (wt %) for purified full-length recombinant AtLEA4-5 (blue) or CS (green). Source Data are provided as a Source Data file.

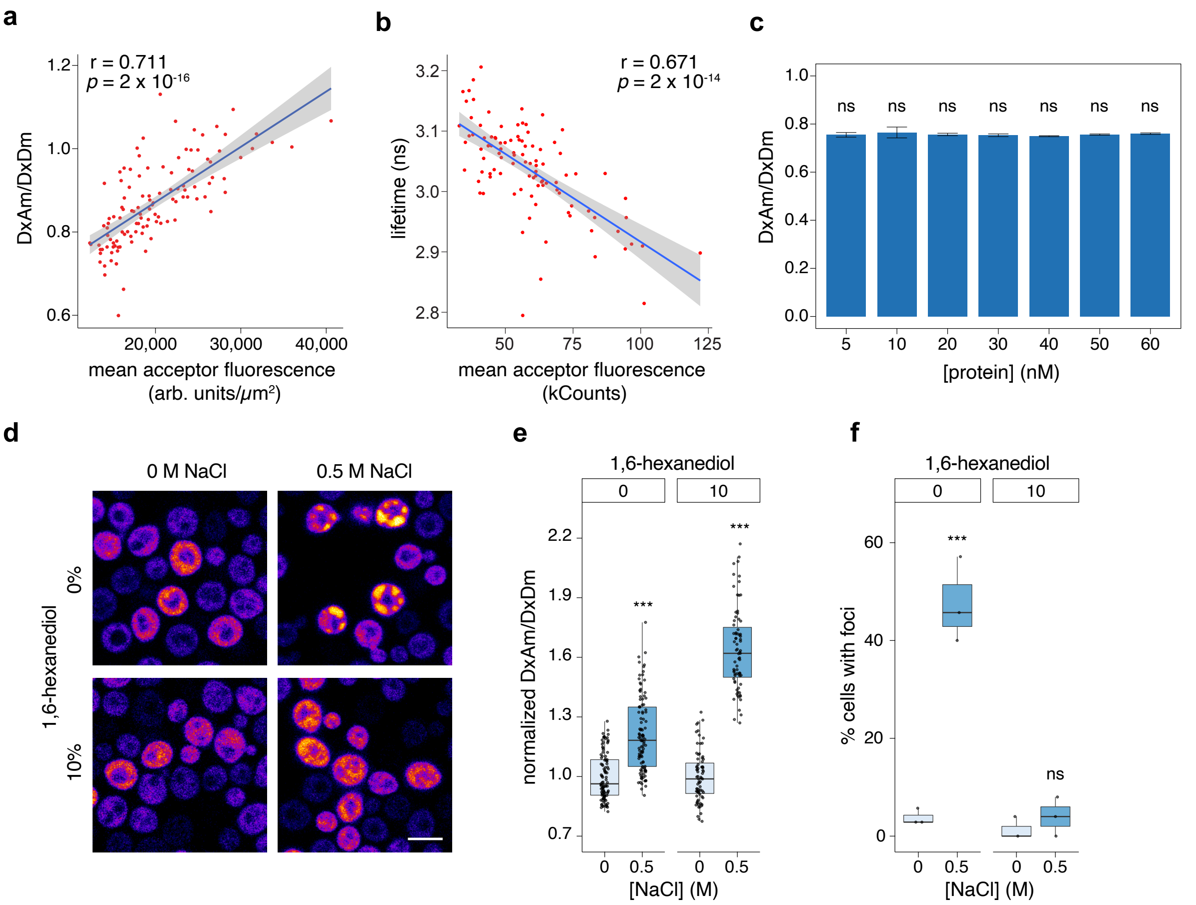

Supplementary Figure 5. (a) Pearson's correlation of FRET ratio (DxAm/DxDm) and mean acceptor fluorescence intensity (arb. units/μm^2) of individual live yeast cells. r = 0.711, p-value = 2×10^{-16}. Continuous line was smoothed using R with a linear method smoothing function. Shaded region indicates 95% confidence interval. (b) Pearson's correlation of fluorescence lifetime of the donor and mean acceptor fluorescence intensity (kCounts) of individual live yeast cells. r = 0.671, p-value = 2×10^{-14}. Continuous line was smoothed using R with a linear method smoothing function. Shaded region indicates 95% confidence interval. (c) FRET ratio (DxAm/DxDm) of purified recombinant full-length SED1 at different concentrations. Mean ± SEM. One-way ANOVA. *p < 0.05, **p < 0.01, ***p < 0.001. (d) Acceptor emission channel with direct acceptor excitation (AxAm) of SED1-expressing live yeast cells under 0 M NaCl and 0.5 M NaCl treatments, in the presence or absence of 10% (wt %) 1,6-hexanediol. Scale bar = 5 μm. The experiment was repeated 3 times independently with similar results. (e) Normalized FRET ratio (DxAm/DxDm) of SED1-expressing live yeast cells under 0 M NaCl and 0.5 M NaCl treatments, in the presence or absence of 10% (wt %) 1,6-hexanediol. n = 3 images with 35 cells per image (0% 1,6-hexanediol) or 25 cells per image (10% 1,6-hexanediol). Two-way ANOVA. ***p < 2 x 10^{-16}. Boxes represent 25th-75th percentile (line at median) with whiskers at 1.5*IQR. (f) Percentage of cells with visible foci in SED1-expressing live yeast cells under 0 M NaCl and 0.5 M NaCl treatments, in the presence or absence of 10% (wt %) 1,6-hexanediol. n = 3 independent images per treatment. Two-way ANOVA. ***p = 2 x 10^{-5}. Boxes represent 25th-75th percentile (line at median) with whiskers at 1.5*IQR. Source Data are provided as a Source Data file.

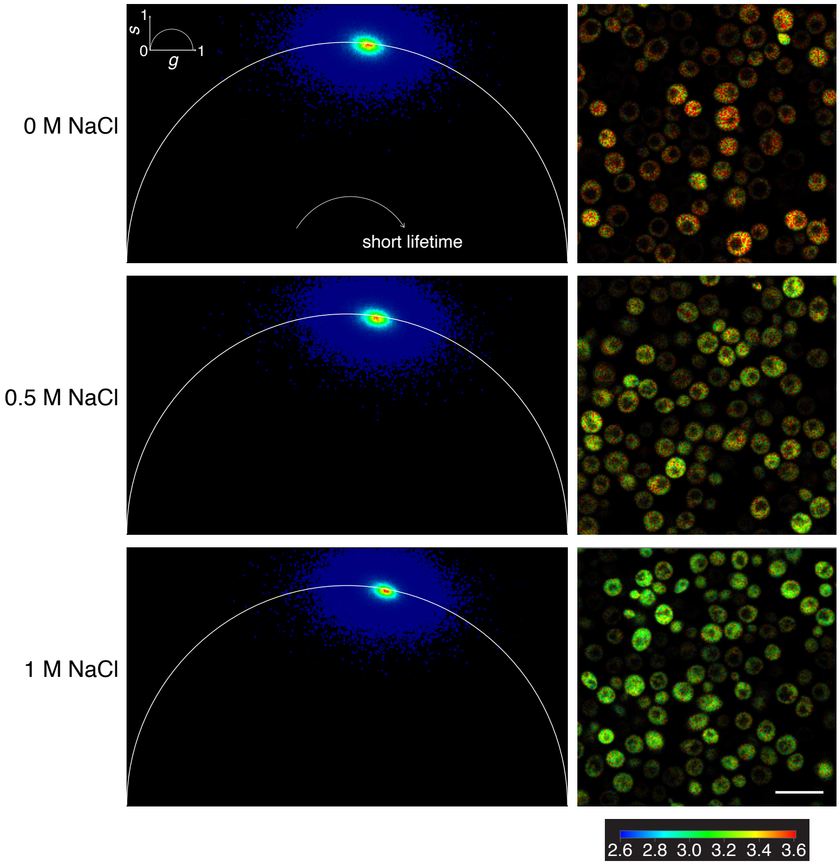

Supplementary Figure 6. Phasor plots (left) and donor fluorescence lifetime images (right) of live yeast cells expressing AtLEA4-5 fused to mCerulean3 (donor-only control) under 0 M, 0.5 M, and 1 M NaCl. Signals shifted to the left side of the phasor plot represent longer fluorescence lifetimes, whereas those shifted to the right side represent shorter fluorescence lifetimes. Scale bar = 10 μm. Calibration bar represents the donor fluorescence lifetime in nanoseconds (ns). The experiment was repeated 3 times independently with similar results. Source Data are provided as a Source Data file.

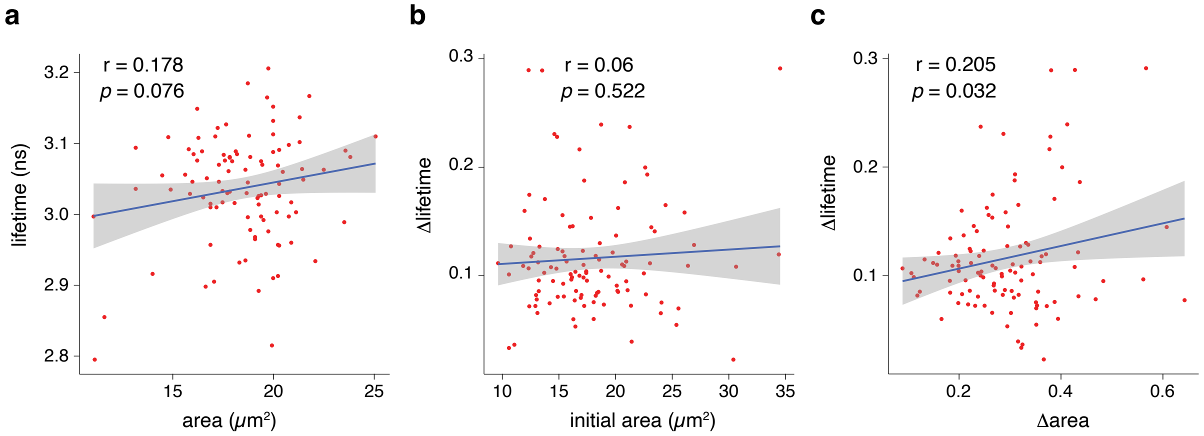

Supplementary Figure 7. (a) Pearson's correlation of donor fluorescence lifetime (ns) and yeast area (proxy of cell volume) under standard conditions (0 M NaCl). r = 0.178, p-value = 0.076. Continuous line was smoothed using R with a linear method smoothing function. Shaded region indicates 95% confidence interval. (b) Pearson's correlation of the change in donor fluorescence lifetime (Δlifetime) and the area (proxy of cell volume) of single yeast cells before treatment with 1 M NaCl. r = 0.06, p-value = 0.522. Continuous line was smoothed using R with a linear method smoothing function. Shaded region indicates 95% confidence interval. (c) Pearson's correlation of the change in donor fluorescence lifetime (Δlifetime) and the change in area (proxy of cell volume) of single yeast cells subjected to 1 M NaCl. r = 0.205, p-value = 0.032. Continuous line was smoothed using R with a linear method smoothing function. Shaded region indicates 95% confidence interval. Δlifetime = (final lifetime - initial lifetime)/initial lifetime. Δarea = (final area - initial area)/initial area. Source Data are provided as a Source Data file.

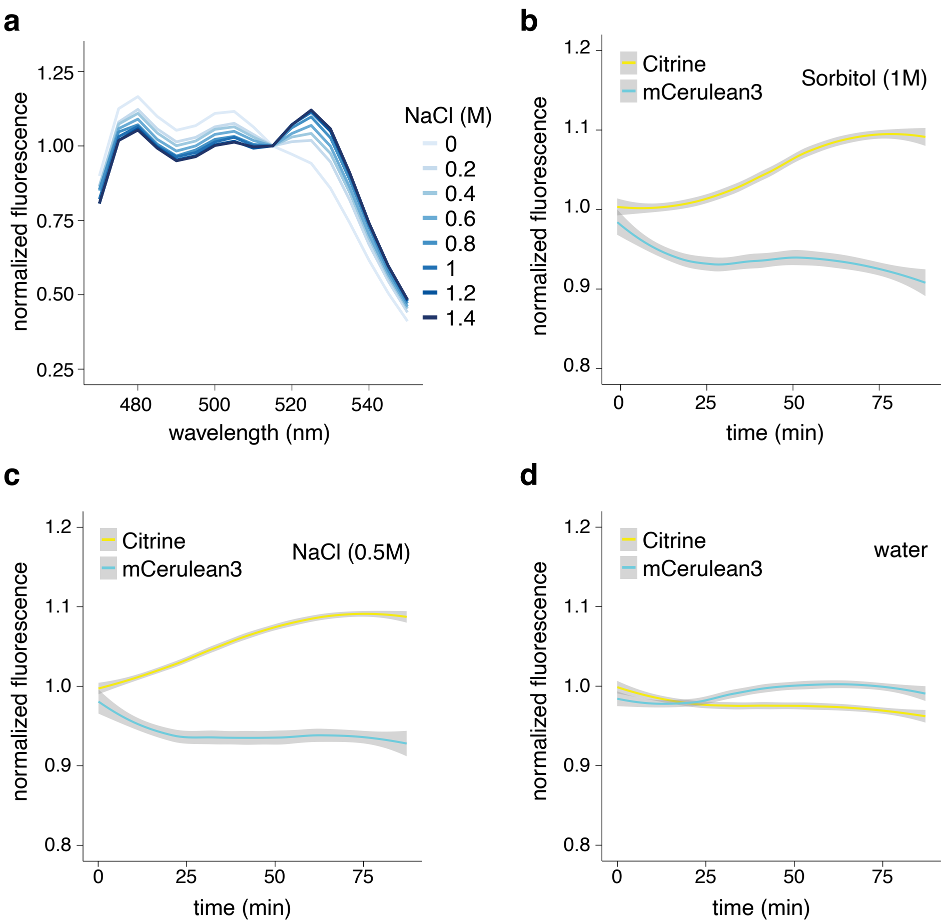

Supplementary Figure 8. (a) Fluorescence emission spectra of NaCl-treated live *Escherichia coli* cells expressing SED1. Fluorescence values were normalized to the value at 515 nm. (b) Normalized fluorescence emission time course of the donor (mCerulean3) and acceptor (Citrine) fluorophores in *Nicotiana benthamiana* leaf discs transiently expressing SED1, treated with 1 M sorbitol. n = 12 leaf discs. Mean ± SEM. One-way ANOVA. (c) Normalized fluorescence emission time course of the donor (mCerulean3) and acceptor (Citrine) fluorophores in *Nicotiana benthamiana* leaf discs transiently expressing SED1, treated with 0.5 M NaCl. n = 12 leaf discs. Mean ± SEM. One-way ANOVA. (d) Normalized fluorescence emission time course of the donor (mCerulean3) and acceptor (Citrine) fluorophores in *Nicotiana benthamiana* leaf discs transiently expressing SED1, treated with pure water. n = 12 leaf discs. Mean ± SEM. One-way ANOVA. Source Data are provided as a Source Data file.

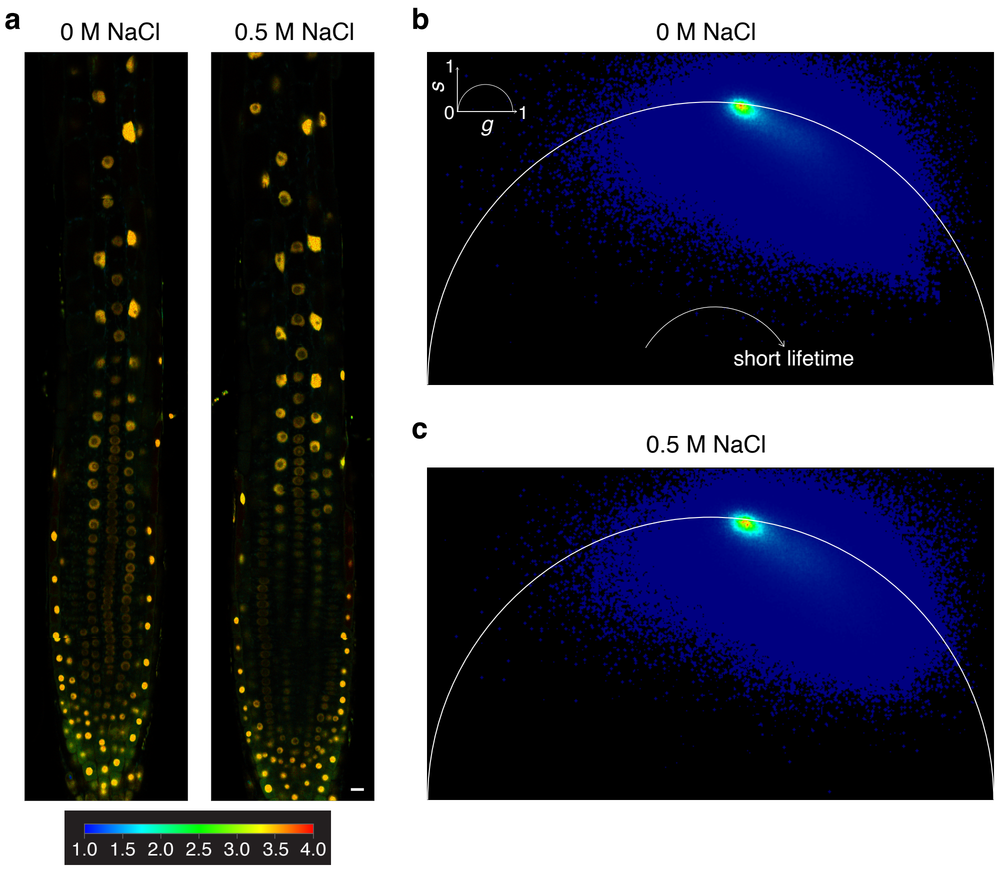

Supplementary Figure 9. (a) Donor fluorescence lifetime images of *pUBQ10::nlsSED1* Arabidopsis roots imaged under 0 M NaCl or 0.5 M NaCl. Signal is localized to nuclei. Scale bar = 10 μm. Calibration bar represents the donor fluorescence lifetime in nanoseconds (ns). The experiment was repeated 3 times independently with similar results. (b) Phasor plot of live *pUBQ10::nlsSED1* Arabidopsis root subjected to 0 M NaCl. (c) Phasor plot of live *pUBQ10::nlsSED1* Arabidopsis root subjected to 0.5 M NaCl. Signals shifted to the left side of the phasor plot represent longer fluorescence lifetimes, whereas signals shifted to the right side represent shorter fluorescence lifetimes. Source Data are provided as a Source Data file.

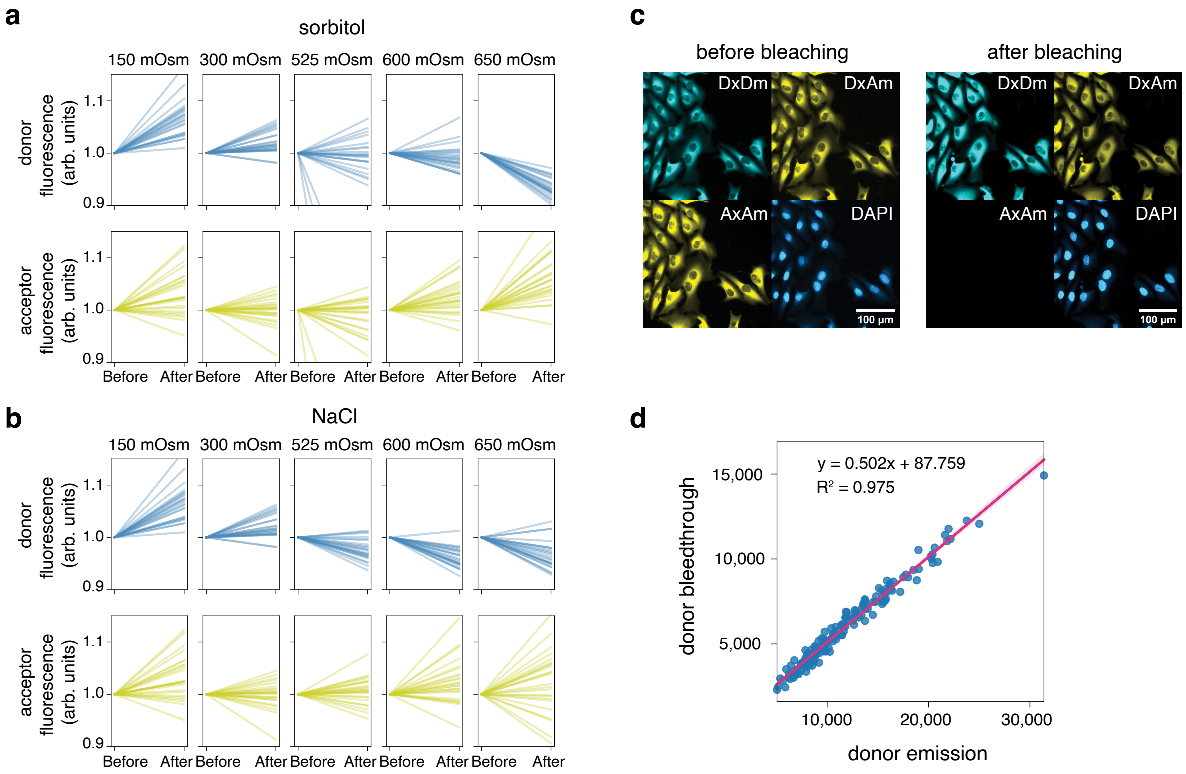

Supplementary Figure 10. (a) Donor and acceptor fluorescence trajectories before and after the treatment of SED1-expressing U-2 OS single cells with sorbitol at the indicated osmolarities. (b) Donor and acceptor fluorescence trajectories before and after the treatment of SED1-expressing U-2 OS single cells with NaCl at the indicated osmolarities. (c) Image sample of live U-2 OS SED1-expressing cells imaged by donor (Dx, 430 nm), acceptor (Ax, 511 nm), or DAPI (385 nm) excitation. To correct for donor bleedthrough, cells were imaged (before bleaching), the acceptor was then photobleached, and the cells were imaged again (after bleaching). The experiment was repeated 3 times independently with similar results. (d) A correlation plot of donor (DxDm) against acceptor (DxAm) emission was used to determine the bleedthrough correction. Multiple wells were imaged and measurements of all cells present in the plain of view were taken from the bleached images. Source Data are provided as a Source Data file.

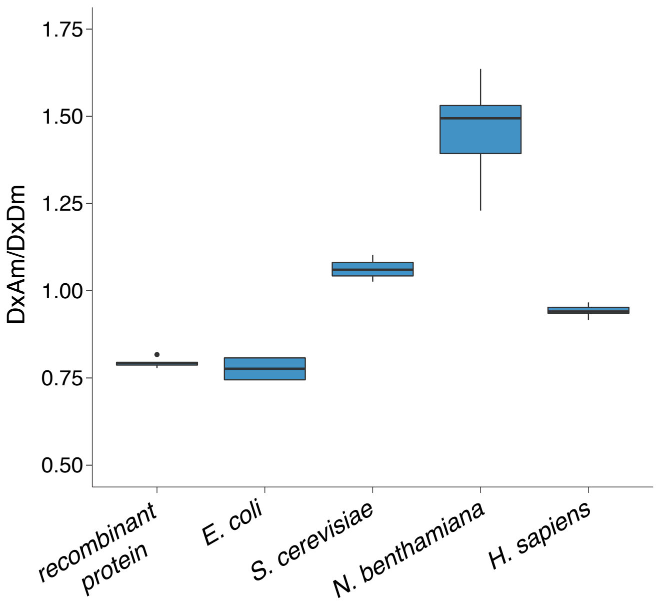

Supplementary Figure 11. (a) FRET ratio (DxAm/DxDm) of SED1 in the indicated context. Purified recombinant full-length SED1 in buffer (20 mM sodium phosphate buffer, 100 mM NaCl, pH 7.4). Standard/isosmotic conditions FRET ratios are shown for the different organisms. n = 12 (recombinant protein); n = 6 (*E. coli*); n = 9 (*S. cerevisiae*); n = 17 (*N. benthamiana*); n = 12 (*H. sapiens*). Boxes represent 25th-75th percentile (line at median) with whiskers at 1.5*IQR. Source Data are provided as a Source Data file.
