# Supplementary material for: Intrinsically disordered protein biosensor tracks the physical-chemical effects of osmotic stress on cells
Source: Nat Commun. 2021 Sep 14;12:5438. doi: 10.1038/s41467-021-25736-8 (PMC8440526; doi:10.1038/s41467-021-25736-8)
Supplement: Supplementary file 3 — Reporting Summary [file 41467_2021_25736_MOESM3_ESM.pdf]

nature research

Corresponding author(s): Cesar Cuevas-Velazquez
Alejandra A Covarrubias
Shahar Sukenik
José Dinneny

Last updated by author(s): Aug 25, 2021

Reporting Summary

Nature Research wishes to improve the reproducibility of the work that we publish. This form provides structure for consistency and transparency in reporting. For further information on Nature Research policies, see our Editorial Policies and the Editorial Policy Checklist.

Statistics

For all statistical analyses, confirm that the following items are present in the figure legend, table legend, main text, or Methods section.

n/a	Confirmed	
☐	☒	The exact sample size (n) for each experimental group/condition, given as a discrete number and unit of measurement
☐	☒	A statement on whether measurements were taken from distinct samples or whether the same sample was measured repeatedly
☐	☒	The statistical test(s) used AND whether they are one- or two-sided<br>*Only common tests should be described solely by name; describe more complex techniques in the Methods section.*
☒	☐	A description of all covariates tested
☒	☐	A description of any assumptions or corrections, such as tests of normality and adjustment for multiple comparisons
☐	☒	A full description of the statistical parameters including central tendency (e.g. means) or other basic estimates (e.g. regression coefficient) AND variation (e.g. standard deviation) or associated estimates of uncertainty (e.g. confidence intervals)
☐	☒	For null hypothesis testing, the test statistic (e.g. F, t, r) with confidence intervals, effect sizes, degrees of freedom and P value noted<br>*Give P values as exact values whenever suitable.*
☐	☒	For Bayesian analysis, information on the choice of priors and Markov chain Monte Carlo settings
☒	☐	For hierarchical and complex designs, identification of the appropriate level for tests and full reporting of outcomes
☐	☒	Estimates of effect sizes (e.g. Cohen's d, Pearson's r), indicating how they were calculated

Our web collection on statistics for biologists contains articles on many of the points above.

Software and code

Policy information about availability of computer code

Data collection	For disorder propensity, we used PONDR web server: http://www.pondr.com/ For alpha-helix prediction, we used AGADIR web server: http://agadir.crg.es/protected/academic/calculation2.jsp For Solution Space Scanning we used the publicly available code https://github.com/sukeniklab/HiddenSensitivity
Data analysis	For data analysis, we used R language and environment (version 4.0.5) and Python programming language. For image analysis we used Fiji: http://fiji.sc/

For manuscripts utilizing custom algorithms or software that are central to the research but not yet described in published literature, software must be made available to editors and reviewers. We strongly encourage code deposition in a community repository (e.g. GitHub). See the Nature Research guidelines for submitting code & software for further information.

Data

Policy information about availability of data

All manuscripts must include a data availability statement. This statement should provide the following information, where applicable:

- Accession codes, unique identifiers, or web links for publicly available datasets
- A list of figures that have associated raw data
- A description of any restrictions on data availability

The authors declare that data supporting the findings of this study are available within the paper and its supplementary information files. Source data are provided with this paper

Field-specific reporting

Please select the one below that is the best fit for your research. If you are not sure, read the appropriate sections before making your selection.

☒ Life sciences ☐ Behavioural & social sciences ☐ Ecological, evolutionary & environmental sciences

For a reference copy of the document with all sections, see nature.com/documents/nr-reporting-summary-flat.pdf

Life sciences study design

All studies must disclose on these points even when the disclosure is negative.

Sample size	Sample sizes used for each experiment is described in the methods sections and figure legends. For all experiments involving cell populations and in vitro samples, sample size is at least n= 4 - 5. For single cell imaging measurements, sample size is at least n = 50.
Data exclusions	No data were excluded from the analysis.
Replication	All experiments were replicated at least 3 times, except for experimental Solution Space Scanning since this experiment involves the characterization of purified recombinant proteins. Experimental solution space scanning was successfully replicated two times. In some cases, replication was performed by more than one author.
Randomization	Randomization was not performed since samples were not allocated to separate experimental groups or clinical trials.
Blinding	Blinding was not done as samples were not allocated to separate experimental groups or clinical trials.

Reporting for specific materials, systems and methods

We require information from authors about some types of materials, experimental systems and methods used in many studies. Here, indicate whether each material, system or method listed is relevant to your study. If you are not sure if a list item applies to your research, read the appropriate section before selecting a response.

Materials & experimental systems

n/a	Involved in the study	
☒	☐	Antibodies
☐	☒	Eukaryotic cell lines
☒	☐	Palaeontology and archaeology
☒	☐	Animals and other organisms
☒	☐	Human research participants
☒	☐	Clinical data
☒	☐	Dual use research of concern

Methods

n/a	Involved in the study	
☒	☐	ChIP-seq
☒	☐	Flow cytometry
☒	☐	MRI-based neuroimaging

Eukaryotic cell lines

Policy information about cell lines

Cell line source(s)	U-2 OS cell line was obtained from ATCC: HTB-96. HEK-293T cell line was obtained from ATCC: CRL-3216.
Authentication	Authentication was performed by ATCC using expression marker and STR profiling.
Mycoplasma contamination	According to ATCC, Mycoplasma contamination was detected and eliminated in 1972 in U-2 OS cell line.
Commonly misidentified lines (See ICLAC register)	No commonly misidentified cell lines were used in the study.
