# Supplementary material for: Intrinsically disordered protein biosensor tracks the physical-chemical effects of osmotic stress on cells
Source: Nat Commun. 2021 Sep 14;12:5438. doi: 10.1038/s41467-021-25736-8 (PMC8440526; doi:10.1038/s41467-021-25736-8)
Supplement: Supplementary file 4 — Description of Additional Supplementary Files [file 41467_2021_25736_MOESM4_ESM.pdf]

Title: Supplementary Movie 1.

Description: Timelapse of donor fluorescence lifetime of single yeast cells expressing SED1 exposed to 1 M NaCl treatment at time 0. Scale bar = 10 μm. Calibration bar is the same as Fig 4c and represents the donor fluorescence lifetime in nanoseconds (ns)".
